# Birth Size in Neonates with Congenital Adrenal Hyperplasia due to 21-hydroxylase Deficiency

**DOI:** 10.4274/jcrpe.galenos.2018.2018.0149

**Published:** 2019-02-20

**Authors:** Helmuth G. Dörr, Theresa Penger, Andrea Albrecht, Michaela Marx, Thomas M. K. Völkl

**Affiliations:** 1 University Hospital of Erlangen, Department of Paediatrics, Division of Paediatrics Endocrinology, Erlangen, Germany

**Keywords:** Term newborn, congenital adrenal hyperplasia, 21-hydroxylase deficiency, genotype

## Abstract

**Objective:**

Classic congenital adrenal hyperplasia (CAH) secondary to 21-hydroxylase deficiency is characterized by increased prenatal adrenal androgen secretion. There are a small number of reports in the literature showing higher birth weight and length in CAH newborns.

**Methods:**

We analyzed birth weight and length data of 116 German newborns (48 boys, 68 girls) with classic CAH who were born during the period from 1990 to 2017. All children have been followed or are currently treated as outpatients in our clinic. All children were born at term. The mothers were healthy and their pregnancies were uneventful. The diagnosis of CAH was confirmed by molecular analyses of the *CYP21A2* gene. Birth data were calculated as standard deviation (SD) scores according to German reference values.

**Results:**

Weight and length in male CAH newborns (mean ± SD) (3601±576 g; 52.4±2.85 cm) were significantly higher than in female CAH newborns (3347±442 g; 51.2±2.55 cm), but male-female differences in the CAH cohort were lost when the data were converted into SD scores. The birth sizes of the CAH newborns did not differ from the reference group. The birth sizes also did not differ between the different CAH genotypes. Maternal age, mode of delivery and maternal parity had no influence on birth size.

**Conclusion:**

Our data show that prenatal hyperandrogenism does not affect fetal growth.

**What is already known on this topic?**

Prenatal weight gain and birth weight are partially influenced by androgen action. Some reports in the literature mention higher birth weight and length in congenital adrenal hyperplasia newborns. However data on this topic are inconsistent.

**What this study adds?**

We studied birth size in term newborns with classic congenital adrenal hyperplasia who were followed in our hospital. Our data support the assumption that prenatal hyperandrogenism has no effect on fetal growth.

## Introduction

Classical congenital adrenal hyperplasia (CAH) is the most common form of inherited disorders of cortisol biosynthesis in the adrenal cortex. It is due to mutations in the active gene *CYP21A2* causing varying degrees of impairment of 21-hydroxylase deficiency (21-OHD) activity. Classical CAH with 21-OHD occurs in two forms, namely, a three-times more frequent form with salt wasting (cortisol + aldosterone deficiency) and a simple virilizing form without aldosterone deficiency (1,2,3). Both forms are characterized by increased adrenal androgen secretion which prenatally causes virilization of the external female genitalia and postnatally, in both sexes if untreated, results in pseudoprecocious puberty, accelerated growth and bone maturation. 

Since term-born male newborns are heavier than females, it has been speculated that prenatal weight gain and birth weight are also at least partially influenced by androgen action (4). In contrast, there are also data showing that birth size is not explained by the effects of prenatal androgen exposure (5).

Some reports in the literature show higher birth weight and length in CAH newborns. According to data from Finland, both girls and boys with classical CAH are significantly longer at birth than healthy newborns of the same ethnic origin (6). Italian authors confirmed these results and speculated that birth size of newborns with classical CAH correlates with the severity of the phenotype (7). In another study from the UK and Sweden, no differences between birth weight standard deviation (SD) scores (SDS) in CAH girls and boys compared with national references and no correlation to the severity of the gene mutation were found (5). Thus the literature contains inconsistent data on this topic.

The objective of our study was to analyse birth weight and length of children with classical CAH who were treated in the outpatient department of our hospital. The severity of the CAH phenotype was determined by molecular genetic classification of the common mutations (8,9,10,11). We attempted to adjust for most factors that might additionally affect birth weight and length.

## Methods

Birth weight and length data of 116 German newborns (48 boys, 68 girls) with classical CAH who were born during the period from 1990 to 2017 were analyzed. All children have been followed or are currently treated as outpatients in our endocrinology clinic. All children were born at term (gestational age: 38 to 41 weeks) either by spontaneous vaginal delivery (n=95) or by caesarean section (n=21). The mothers (age: 20 to 42 years) were healthy primipara (n=77) or multipara (n=39), and the pregnancies were uneventful. Data on maternal body mass index at delivery were not available. 

The diagnosis of CAH was confirmed by molecular analyses of the *CYP21A2* gene. Molecular genetic classification of the severity of CAH was performed according to Krone et al (8) as follows. The genotype ‘Null’ included patients with biallelic mutations that resulted in completely inactive enzymes (e.g. gene deletions), genotype A included patients with homozygous I2G or heterozygous I2G in trans with a null mutation, and B patients with homozygous p.I173N mutation or heterozygous p.I173N mutation in trans with a mutation from group ‘Null’ or group A. Genotype ‘Null’ was found in 43 children, genotype A in 51 children, and 22 children were identified with genotype B. 

Birth weight (g) and length (cm) data were obtained from the patient records (“Vorsorgeheft”). As birth weight we used the weight measured at birth and for length we used the data obtained between three and seven days after birth. The length measurement at that age is part of the clinical examination of the newborn, usually before discharge from the hospital and the value obtained is more reliable than the length measured at birth. Birth data were calculated as SDS according to German reference values as follows (12): SDS = (patient’s measured value-mean value for age- and sex-matched normal subjects) ÷ SD of the values for age- and sex-matched normal subjects. The German reference data used are based on the perinatal data of 2.3 million singleton newborns from 1995-2000 (12). We defined all neonates with a birth weight and length of <-2 SDS as small for gestational age (SGA) and with the same parameters, those with measurements of >2 SDS as large for gestational age (LGA). 

The study design (retrospective analysis of the data) was approved by the Ethical Committee of our Hospital without an approval number. Informed consent has been obtained from the parents after full explanation of the purpose and nature of all procedures used.

### Statistical Analysis

Statistical analysis was performed using SPSS, Version 21 (IBM Inc., Chicago, Ill., USA). Data are expressed as mean ± SD and median. Kruskal Wallis test was used to compare values between different genetic groups. Student t-test for unpaired samples was used to compare weight and length values between status of maternal parity and mode of delivery.

## Results

Birth size (weight and length) in term newborns with classic CAH are shown in Tables 1 and 2. We found no statistically significant difference between the birth size of the CAH newborns compared with the reference group. In terms of birth weight, three children of the CAH cohort were classified as SGA and six as LGA newborns. Mean birth size (weight in grams and length in centimeters) in male CAH newborns was statistically significantly higher than in females (weight: p<0.01; length p<0.02). However, when comparing the data after SDS conversion, neither weight-SDS nor length-SDS values were significantly different between the two sexes. Birth size was also not different between the different CAH genotypes. We analyzed the data also according to genotype and sex and found no difference. Moreover, maternal age, mode of delivery and maternal parity had no influence on birth size.

## Discussion

Androgen action on birth size is implicated by the fact that healthy male newborns are longer and weigh more than female newborns (13). Children with classical CAG and 21-OHD (CAH) have increased adrenal androgen secretion that prenatally causes virilization of the external female genitalia. Additionally, there are some reports in the literature showing that prenatal hyperandrogenism also affects birth size in CAH newborns. 

In 1971, a study from Canada compared the birth weights of CAH newborns with their unaffected siblings and normal newborns and found that only females with CAH were heavier than the female controls and female siblings (14). Jaaskelainen and Voutilainen (6) from Finland reported that both girls and boys with classic CAH are significantly longer at birth than healthy newborns of the same ethnic origin. The authors did not make any distinction between the different clinical forms of classic CAH. 

In addition Italian authors reported that the mean birth length in both boys and girls with classical CAH was significantly greater than the mean birth length in healthy Italian children and speculated that the birth data correlated with the severity of the phenotype (7). In contrast data from the UK and Sweden showed no differences between birth weight SDS in CAH girls and boys in relation to the national references and no correlation to the severity of the gene mutation (5). In a study from Munich, mean birth “height” SDS data of 51 newborns with classical CAH, diagnosed by newborn screening, was found to be slightly above average (15). Chalmers et al (16) identified 105 CAH newborns over a long period of 50 years and found no difference in birth weight from the standard population median and also no sex difference in favour of heavier males. They speculated that these differences were ameliorated because of increased levels of prenatal androgens experienced by the female infants. 

Furthermore, in a large retrospective observational cohort study from France, heavier male than female CAH newborns, but overall normal mean birth weight and birth length were reported (17). Our data confirm this sex-related difference. In our analysis, male CAH newborns had statistically significant higher birth weight (g) and length (cm) values than females. When the data were transformed to SDS values, the male newborns still had slightly higher values than the females, but the difference was not statistically significant. Birth sizes of our cohort were not different from the German reference population. The severity of CAH, maternal age, parity and mode of delivery had no influence on birth size. In terms of birth weight, three children in our cohort were classified as SGA and six as LGA newborns.

It is not surprising that the results in the literature are inconsistent. Most reports on birth size in CAH newborns do not provide data on the course of pregnancy, maternal age, maternal parity status, or mode of delivery. The somatic classification of neonates is primarily based on birth weight. Birth weight is affected by a multitude of different factors such as socio-economic status, maternal age and concomitant diseases of the mother in pregnancy, as well as placental, fetal and environmental conditions (18,19,20,21). Additionally, the correct interpretation of birth sizes is complicated by the methodological heterogeneity and limitations of birth size charts available worldwide. In addition secular trends in birth size over the last 25 year period might play a role. Consequently, there are numerous factors that limit the interpretation of birth size in CAH newborns.

### Study Limitations

There were some limitations to our study. The sample size is too small to exclude a type 2 statistical error. The study is retrospective. The data analyzed cover a period from 1990 to 2017. It was not possible to exclude all the different factors which might affect birth size of newborns with CAH.

## Conclusions

In general data on birth size of newborns with CAH secondary to 21-OHD are scarce. We tried to clarify existing conflicting published data on this topic. Our data support the assumption that prenatal hyperandrogenism has no effect on fetal growth.

## Figures and Tables

**Table 1:**
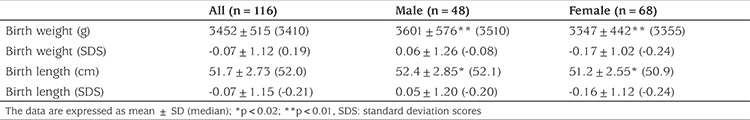
Birth size in term male and female newborns with classical congenital adrenal hyperplasia. P values refer to comparison between the genders

**Table 1:**
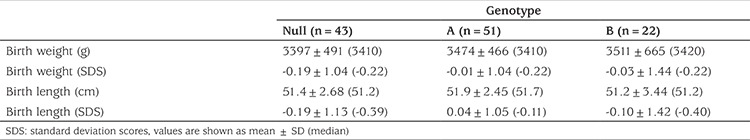
Birth size in newborns with classical congenital adrenal hyperplasia according to genotype
